# Ototoxicity of polystyrene nanoplastics in mice, HEI-OC1 cells and zebrafish

**DOI:** 10.3389/fnmol.2024.1345536

**Published:** 2024-02-19

**Authors:** Yuancheng Wu, Lianzhen Li, Lihuan Tang, Willie Peijnenburg, Huangruici Zhang, Daoli Xie, Ruishuang Geng, Tihua Zheng, Liyan Bi, Xiaodan Wei, Han-jung Chae, Lan Wang, Li Zhao, Bo Li, Qingyin Zheng

**Affiliations:** ^1^Hearing and Speech Rehabilitation Institute, Binzhou Medical University, Yantai, China; ^2^College of Environmental Sciences and Engineering, Qingdao University, Qingdao, China; ^3^Institute of Environmental Sciences (CML), Leiden University, Leiden, Netherlands; ^4^National Institute of Public Health and the Environment (RIVM), Center for Safety of Substances and Products, Bilthoven, Netherlands; ^5^Department of Pathology, School of Basic Medicine, Binzhou Medical University, Yantai, China; ^6^School of Pharmacy, Jeonbuk National University, Jeonju, Republic of Korea; ^7^Department of Otolaryngology-Head and Neck Surgery, Case Western Reserve University, Cleveland, OH, United States

**Keywords:** polystyrene nanoplastics, ototoxicity, hearing loss, environmental ototoxicants, reactive oxygen species, inflammation, ferroptosis

## Abstract

Polystyrene nanoplastics are a novel class of pollutants. They are easily absorbed by living organisms, and their potential toxicity has raised concerns. However, the impact of polystyrene nanoplastics on auditory organs remains unknown. Here, our results showed that polystyrene nanoplastics entered the cochlea of mice, HEI-OC1 cells, and lateral line hair cells of zebrafish, causing cellular injury and increasing apoptosis. Additionally, we found that exposure to polystyrene nanoplastics resulted in a significant elevation in the auditory brainstem response thresholds, a loss of auditory sensory hair cells, stereocilia degeneration and a decrease in expression of Claudin-5 and Occludin proteins at the blood-lymphatic barrier in mice. We also observed a significant decrease in the acoustic alarm response of zebrafish after exposure to polystyrene nanoplastics. Mechanistic analysis revealed that polystyrene nanoplastics induced up-regulation of the Nrf2/HO-1 pathway, increased levels of malondialdehyde, and decreased superoxide dismutase and catalase levels in cochlea and HEI-OC1 cells. Furthermore, we observed that the expression of ferroptosis-related indicators GPX4 and SLC7A11 decreased as well as increased expression of ACLS4 in cochlea and HEI-OC1 cells. This study also revealed that polystyrene nanoplastics exposure led to increased expression of the inflammatory factors TNF-α, IL-1β and COX2 in cochlea and HEI-OC1 cells. Further research found that the cell apoptosis, ferroptosis and inflammatory reactions induced by polystyrene nanoplastics in HEI-OC1 cells was reversed through the pretreatment with N-acetylcysteine, a reactive oxygen species inhibitor. Overall, our study first discovered and systematically revealed the ototoxicity of polystyrene nanoplastics and its underlying mechanism.

## 1 Introduction

Since the 1950s, the production and consumption of plastics have increased rapidly worldwide, resulting in the generation of large quantities of plastic waste. Currently, over 400 million tons of plastics are produced each year (Ain Bhutto and You, 2022). This leads to substantial accumulation in terrestrial and aquatic ecosystems. According to their size, plastics found in the environment could be divided into three main groups: nanoplastics (NPs, 1 to <1000 nm), microplastics (MPs, 1 to <5000 μm) and macroplastics (≥5 mm) (Gigault et al., 2018; Frias and Nash, 2019). Plastic pollution has become an increasingly global ecological threat in recent years and especially MPs and NPs are increasingly recognized as hazards to the environment (Allouzi et al., 2021). Further evidence has shown that MPs/NPs are detected in water and aquatic products, as well as in food crops. MPs/NPs could therefore be easily ingested by humans and animals through drinking water and via the diet, among other routes (Yang D. et al., 2022). Indeed, there are studies that have identified the presence of MPs/NPs in human feces, lungs, and even blood (Schwabl et al., 2019; Jenner et al., 2022; Leslie et al., 2022). The existence of MPs/NPs in the body may lead to inconceivable health impacts on humans and animals (Ullah et al., 2022). Due to their minuscule size and poor biodegradation of NPs, it is easy for organisms to ingest and accumulate NPs for a long time. In fact, NPs could penetrate cell membranes to access lymph nodes and the blood circulation, subsequently accumulating in multiple tissues and organs of animals, and potentially causing adverse effects on physiological functions (Sun et al., 2022). Therefore, it is crucial to conduct studies on the toxicity of NPs. The main chemical components of NPs include polystyrene (PS), polyethylene (PE), and polypropylene (PP). PS is a commonly utilized plastic in both daily life and industrial settings due to its advantageous characteristics of cost-effectiveness, durability, and moisture proofness. However, the Polystyrene nanoplastics (PS-NPs) are exceedingly difficult to biodegrade, and they could be permanently stable in the environment (Ho et al., 2018).

Numerous investigations have reported that PS-NPs induce toxic effects on different organs among several organisms such as mice and zebrafish. One study has found that PS-NPs suppressed cell proliferation, promoted apoptosis, and enhanced autophagy to produce cytotoxicity (Ding et al., 2021). Further studies have reported that PS-NPs accumulate in the spleen, liver, lungs, kidneys, testes, small and large intestine, and brains of mice following oral ingestion of PS-NPs (Choi et al., 2021; Xu et al., 2021). This accumulation has resulted in nephrotoxicity, hepatotoxicity, neurotoxicity and abnormal lipid metabolism in mice (Fan et al., 2022; Meng et al., 2022; Shan et al., 2022). Choi et al. (2021) observed toxic effects of PS-NPs on mice, including oxidative stress, autophagy, inflammatory response, and apoptosis. Qiu et al. (2023) have shown that co-exposure to cadmium and PS-NPs triggers oxidative damage through the Nrf2/HO-1 signaling pathway. In addition, PS-NPs are capable of accumulating in and posing a threat to aquatic organisms (Wang et al., 2022). Due to the advantages of zebrafish in toxicological research, such as small body size, low cost, high fecundity, and genetic similarity to humans, many researchers have attempted to evaluate the accumulation and toxicity of PS-NPs targeting zebrafish (Bhagat et al., 2020). Several investigators have suggested that PS-NPs may impact the reproductive capacity of zebrafish and induce toxic effects such as hepatotoxicity, neurotoxicity and harmful cardiovascular effects on these aquatic organisms (Pedersen et al., 2020; Sun et al., 2021; Kim et al., 2022; Park and Kim, 2022).

This current paper targets the effects of PS-NPs on the auditory systems of various organisms such as mice, HEI-OC1 cells and zebrafish. A recent *in vitro* study revealed that co-cultured cochlear explants from neonatal mice exposed to a low concentration of PS-NPs, showed intracellular entry of PS-NPs (Nikolic et al., 2022). This suggests a potential threat of PS-NPs to the auditory system of animals. However, whether PS-NPs can accumulate in cochlea and affect the function of the auditory system has not been investigated. Current data suggested that approximately 5% of the world’s population suffer from disabling hearing loss, which greatly affects their work and life (Torrente et al., 2023). Ototoxic substances are one of the leading causes of hearing loss (Fabelova et al., 2019). Therefore, further studies are required to clarify the potential toxic impacts of PS-NPs on the auditory system. Except in mouse experiments, the HEI-OC1 cell line is one of the most commonly used mouse auditory cell lines that was initially proposed as an *in vitro* screening system for ototoxic drugs, well suited for investigating the molecular mechanism of ototoxicity of PS-NPs (Kalinec et al., 2016). PS-NPs pose health hazards to mammals. In addition to this, the impacts of PS-NPs on aquatic animals must also be taken into consideration. The auditory organs of zebrafish, known as lateral line neuromasts, are distributed on the outer surface of zebrafish to detect localized water motion. Zebrafish lateral line sensory hair cells are structurally and functionally homologous to mammalian hair cells, and are widely utilized as a valuable model system for investigating hair cell injury (Holmgren et al., 2021).

## 2 Materials and methods

### 2.1 Characterization of PS-NPs

In this study, we utilized two types PS-NPs with similar size and shape. One type was yellow-green fluorescent PS-NPs (size = 30 nm, λ_ex_ = 470 nm, λ_em_ = 505 nm, Sigma Aldrich, USA), the fluorescent dyes are embedded in the beads without any chemical connections to maintain the stability of the fluorescence of PS-NPs. These fluorescent PS-NPs are carboxylate modified, in which the carboxyl groups are negatively charged. Another type was pristine PS-NPs (size = 25 nm, Bangs Laboratories, IN, USA), which do not contain fluorescent dyes or any groups. In our study, the fluorescent PS-NPs were used to observe and locate the distribution of PS-NPs in the auditory organs. To prevent fluorescent dyes from affecting the toxicological experiments, pristine PS-NPs were used for conducting toxicity studies. We examined the morphology of these two types of PS-NPs using transmission electron microscopy (JEM-1400, Japan) and scanning electron microscopy (Zeiss ULTRA-plus, Germany), and assessed the zeta potential and particle size of these PS-NPs performed on Zeta Sizer Nano ZS (Malvern Zetasizer 3000HS, UK).

### 2.2 Animal care

C57BL/6 male mice (6 weeks of age) were purchased from the Nanjing model animal research center (Nanjing, China) and housed in a temperature (20°C) and humidity controlled (40–70%) with a 12 h light/12 h dark cycle pathogen-free mice facility (Xie et al., 2021). These mice were randomly divided into three groups: a control group (Con), a low-dose PS-NPs group (LD), and a high-dose PS-NPs group (HD). The optimal dosage for PS-NPs administration used in the animals was chosen based on previous studies on tissue accumulation, effects of orally administered PS-NPs on toxicity, and inflammatory response in mice (Deng et al., 2017; Choi et al., 2021). C57BL/6 mice in each group were orally administered the same amount of purified water (Con), 5 mg/kg PS-NPs (LD) or 25 mg/kg PS-NPs (HD) for 8 consecutive weeks. C57BL/6 mice had *ad libitum* access to food.

Zebrafish Embryos (AB strain) were purchased from Shanghai FishBio Co., Ltd (Shanghai, China). Zebrafish Embryos were cultivated in an incubator at a suitable temperature (29°C) using embryo medium (EM) containing 5 mM NaCl, 0.33 mM CaCl_2_, 0.17 mM KCl, and 0.33 mM MgCl_2_, with a 14 h light/10 h dark cycle (Wilson, 2012). Zebrafish were randomly divided into 3 groups at 5 days post-fertilization (dpf). The zebrafish in the control group were raised in normal EM, while the other two groups were raised in EM containing 0.005 mg/mL PS-NPs (LD group) and 0.05 mg/mL PS-NPs group (HD group), respectively, for 120 h. The Animal Use and Care Committee of Binzhou Medical University approved this experimental protocol, and all animals were treated humanely to reduce suffering.

### 2.3 Auditory brainstem response

Auditory brainstem responses (ABR) were recorded for three groups of mice at the age of 6, 10 and 14 weeks using BioSigRZ software from Tucker Davis Technologies (TDT, FL, USA). C57BL/6 mice were anesthetized with tribromoethanol (200 mg/kg) and placed in a sound attenuating chamber. Additionally, a constant temperature heating blanket was used to maintain body temperature of mice. The active electrode was inserted subcutaneously over the vertex of the skull. The reference and ground electrode were inserted subcutaneously behind the right and left ears, respectively. ABR was evoked by different frequencies of sound (clicks, 8, 16, and 32 kHz) through the closed-field speaker. As the lowest level at which a visual waveform response could be observed, the ABR threshold was defined (Liu et al., 2023).

### 2.4 Measurement of the accumulation and distribution of fluorescent PS-NPs

Accumulation of fluorescent PS-NPs in the cochlea was detected using the *in vivo* imaging system (Aniview, China) with a 465 nm excitation filter and 520 nm emission filter, as previously described (Yang D. et al., 2022). To observe the distribution of fluorescent PS-NPs in the cochlea of mice, cochlear tissues were sliced into 5 μm sections and observed under fluorescence confocal microscopy (LSM 880, Zeiss, Germany). The presence of PS-NPs in the cochlea was indicated by yellow-green fluorescence. In addition, the accumulation and distribution of fluorescent PS-NPs in HEI-OC1 cells and zebrafish lateral line hair cells was directly observed under confocal microscopy.

### 2.5 Electron microscopy

To prepare for scanning electron microscopy, cochleae were fixed using 2.5% glutaraldehyde, as previously described (Zhao et al., 2021a). After rinsing with 0.01 M PBS, the bone, the spiral ligament, and the Reissner’s membrane were dissected. Subsequently, tissues were fixed with 1% osmium tetroxide for 1.5 h. After ethanol dehydration, the basilar membrane was coated with gold palladium. The observation of the basilar membrane was carried out using high-resolution scanning electron microscopy (Zeiss ULTRA-plus, Germany).

### 2.6 Western blotting

The whole cochlea was harvested for experiments immediately after the cessation of PS-NPs treatment at 14 weeks of age. Tissues and cells were lysed and extracted with Radio Immunoprecipitation Assay (RIPA, Thermo Fisher Scientific, CA, USA) (Zhao et al., 2021b). Equal amounts of protein extracts of cochlear tissues (40–70 μg) and HEI-OC1 cells (30–50 μg) were transferred onto a polyvinylidene difluoride membrane (PVDF, Merck Millipore, MO, USA) following sodium salt-polyacrylamide gel electrophoresis (SDS-PAGE). The membranes were blocked with a non-protein fast blocker (Shandong Hichen Biotechnology Co., Ltd, China) for 5 min at room temperature, and were then probed overnight at 4°C with relevant primary antibodies (Supplementary Table 1). The membranes were probed again for 1 h at room temperature with a species-specific secondary antibody coupled to horseradish peroxidase, after washing with Tris-Buffered Saline-Tween 20 (TBS-T). The Chemiluminescent HRP Substrate kit (Merck Millipore, USA) was used to detect the Western blots bands, and the bands were visualized using a Chemidoc XRS + system (Bio-Rad, USA). The intensity of the protein bands was measured and quantified with ImageJ software from the National Institutes of Health.

### 2.7 Immunofluorescence

The whole cochlea was harvested for experiments immediately after the cessation of PS-NPs treatment at 14 weeks of age. After deparaffinization, rehydration, the sections were incubated with an anti-Occludin antibody, anti-Claudin 5 antibody and DAPI as described elsewhere (Yan et al., 2022). The stained sections were imaged using confocal fluorescence microscopy.

### 2.8 HEI-OC1 cell culture

The HEI-OC1 cell line was provided by Professor Federico Kalinec (House Ear Institute, CA, USA) and is a widely used auditory cell line in mice. As described previously (Zhao et al., 2021b), the HEI-OC1 cells were cultured under appropriate conditions (33°C, 10% CO_2_) and supplemented with 10% fetal bovine serum (FBS; Gibco, CA, USA) without any antibiotics. HEI-OC1 cells were exposed to 0, 50, 100, 200, and 500 μg/mL PS-NPs for 24 h in a cell incubator. For inhibitor pretreatment groups, the reactive oxygen species (ROS) inhibitor N-acetylcysteine (NAC, 5 mM. Selleck, TX, USA) was added to HEI-OC1 cells for 2 h at 33°C, and the HEI-OC1 cells were then exposed to the IC50 concentrations of PS-NPs for 24 h at 33°C.

### 2.9 Real time cell analysis (RTCA)

Cytotoxicity was evaluated with the xCELLigence RTCA DP system (ACEA Biosciences, CA, USA) as described (Zhao et al., 2021b). RTCA is a technology leveraging the use of impedance and microsensor electrodes. The electronic impedance of the sensor electrodes facilitates detection of the attachment of cells on the wells’ bottom, with cell spreading monitored and expressed as the cell index value. The cell index value is defined as (Zi–Z0 Ω)/15 Ω, where Z0 is the background impedance of the well measured without cells (medium alone) and Zi is the impedance of the well measured at any time with cells present (Stefanowicz-Hajduk and Ochocka, 2020). The background of E-plates was determined in 50 μL of medium to establish background signals, followed by addition of 100 μL of HEI-OC1 cell suspension at a density of 1.3 × 10^4^ cells per well. After incubation for 30 min at room temperature, the E-plates were placed in the RTCA station and the HEI-OC1 cells were monitored for at least 24 h, with impedance measurements taken every 15 min. Following the designated treatments, impedance measurements were taken every 15 min until the end of the experiment. The cell-sensor impedance, reflecting the growth of adherent cells and measured as the cell index, was recorded as an arbitrary unit by the RTCA software and normalized at the selected time point immediately before the addition of treated drugs. Each treatment was performed in triplicate.

### 2.10 Cell counting kit-8 (CCK-8) cell viability assay

The HEI-OC1 cells were seeded in a 96-well plate at a density of 1 × 10^5^/mL. Different concentrations (0, 50, 100, 200, 500 μg/mL) of PS-NPs were added to the 96-well plates after the cells were attached. Cell viability was measured using the CCK-8 (Meilun Biotechnology, Dalian, China) according to the manufacturer’s instructions after culture for 24 h.

### 2.11 Biochemical evaluation

The protein concentration of cochlear tissue and HEI-OC1 cells was measured employing an enhanced BCA protein assay kit (Beyotime Biotechnology, Shanghai, China). The activities of superoxide dismutase (SOD), catalase (CAT), and the malondialdehyde (MDA) content were measured using commercial reagent kits obtained from Beyotime Biotechnology according to the specification and all procedures were conducted at 4°C.

### 2.12 Hair cell labeling in zebrafish

FM1-43 (N-(3-Triethylammoniumpropyl)-4-(4-(Dibutylamino) Styryl) Pyridinium Dibromide, Thermo Fisher, CA, USA) was used to label hair cells of zebrafish. Larvae were anesthetized with tricaine methanesulfonate and exposed to a 3 μM concentration of FM1-43 for 15 s. Subsequently, they were mounted on a FluoroDish with low-melt agarose that contained 0.01% tricaine. The agarose was allowed for 2.5 min to solidify before being imaged.

### 2.13 TUNEL staining

In order to label and quantify apoptotic cells in lateral line neuromasts of zebrafish larvae, TUNEL (terminal deoxynucleotidyl transferase-mediated dUTP nick-end labeling) staining was conducted on zebrafish after treatment with PS-NPs for 120 h. Larvae were first incubated in 0.01 M PBS solution for 10 min and then rinsed three times with PBS during 10 min. Then, the permeabilization step was carried out using 0.1% Triton X-100, and the nuclei were counterstained with DAPI. Larvae were processed using the TUNEL cell apoptosis detection kit (Roche, Switzerland) following the instructions provided by the manufacturer.

### 2.14 Acoustic startle response test

Acoustic startle response test was generated using the DanioVision Tapping Device (Noldus, Gelderland, Netherlands), which produces acoustic vibrations (Faria et al., 2019). Zebrafish larvae were individually placed in 96-well plates containing 200 μL of EM. Prior to the acoustic stimulation, the locomotion of the zebrafish was meticulously documented for a duration of 1 s, followed by an additional 2 s of continuous recording. Ethovision software 16 was used to analyze the zebrafish’s distance moved, maximum velocity and maximum acceleration every 0.2 s during the 3 s.

### 2.15 Statistical analysis

Each group of mice was randomized and each trial was conducted at least 3 times. Statistical analysis was performed with GraphPad Prism 9 (GraphPad software, CA, USA). The data is presented as mean ± standard deviation. One-way analysis of variance (ANOVA) was employed to test significant differences among more than two groups. *P*-values that were smaller than 0.05 indicated significant differences.

## 3 Results

### 3.1 Characterization of the PS-NPs

**FIGURE 1:**
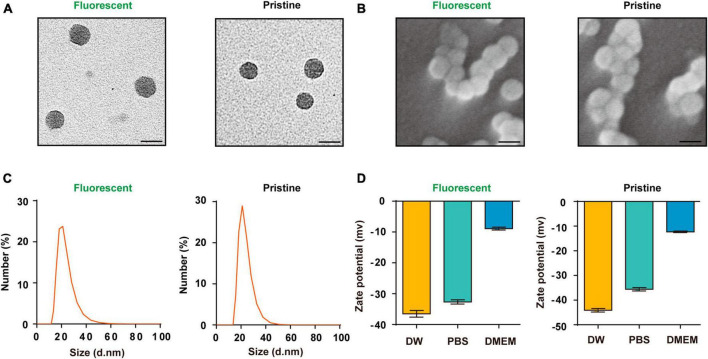
Characterization of the PS-NPs. **(A,B)** Transmission electron microscopy images of fluorescent PS-NPs and pristine PS-NPs, scanning electron microscopy images of fluorescent PS-NPs and pristine PS-NPs. **(C)** Hydrodynamic size distribution of fluorescent PS-NPs and pristine PS-NPs determined by Dynamic Light Scattering (DLS) analysis. **(D)** Zeta potential value of fluorescent PS-NPs and pristine PS-NPs dispersed in Distilled Water (DW), PBS, and DMEM. Error bars represent SEM.

The PS-NPs that were used in this study had a spherical shape, as observed from transmission electron microscopy and scanning electron microscopy analyses (Figures 1A, B). Dynamic light scattering analyses revealed that the hydrodynamic diameter dimension of the fluorescent PS-NPs is 20.4 ± 0.8 nm, the diameter of the pristine PS-NPs is 21.9 ± 0.6 nm (Figure 1C). Zeta potential is an important index to characterize the stability of colloidal dispersion system (Bhattacharjee, 2016). The zeta potential of fluorescent PS-NPs in DW, PBS and DMEM is, respectively, −36.5 ± 1.9, −32.7 ± 1.2, and −8.9 ± 0.8. The zeta potential of pristine PS-NPs in DW, PBS, and DMEM is, respectively, −43.43 ± 0.06, −35.6 ± 1.8, and −12.3 ± 0.5 (Figure 1D).

### 3.2 Cochlear absorption of PS-NPs causes hearing loss in mice

**FIGURE 2:**
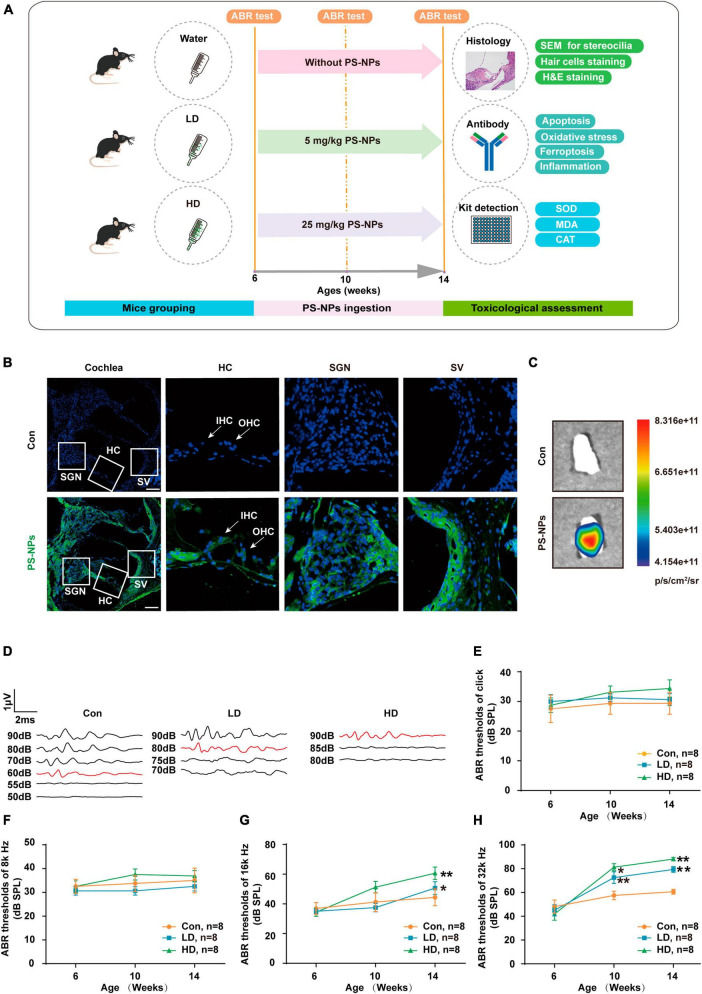
Polystyrene-NPs are absorbed by cochlea tissues and elevate ABR thresholds in mice. **(A)** Flow chart diagram of the mice experiments. **(B)** Distribution of fluorescent PS-NPs in the cochlea of mice observed by fluorescence microscopy, the green color indicates fluorescent PS-NPs, and the nuclei are blue (stained by DAPI) (Scale bar = 200 μm). **(C)** Bioaccumulation of fluorescent PS-NPs in the inner ear of mice was analyzed by an animal *in vivo* imaging system. **(D)** Representative serial ABR wave recordings. Comparison of the average ABR thresholds of mice in Control, L-Dose, H-Dose groups for clicks **(E)**, 8 k Hz **(F)**, 16 k Hz **(G)**, and 32 k Hz **(H)** at 6, 10, and 14 weeks (Number of each group, *n* = 8). The ABR thresholds in the LD, and HD groups were compared with those in the con group, *p < 0.05, **p < 0.01. Error bars represent SEM. Con: control group, 0 mg/kg PS-NPs; LD: low dose group, 5 mg/kg PS-NPs; HD: high dose group, 25 mg/kg.

**FIGURE 3:**
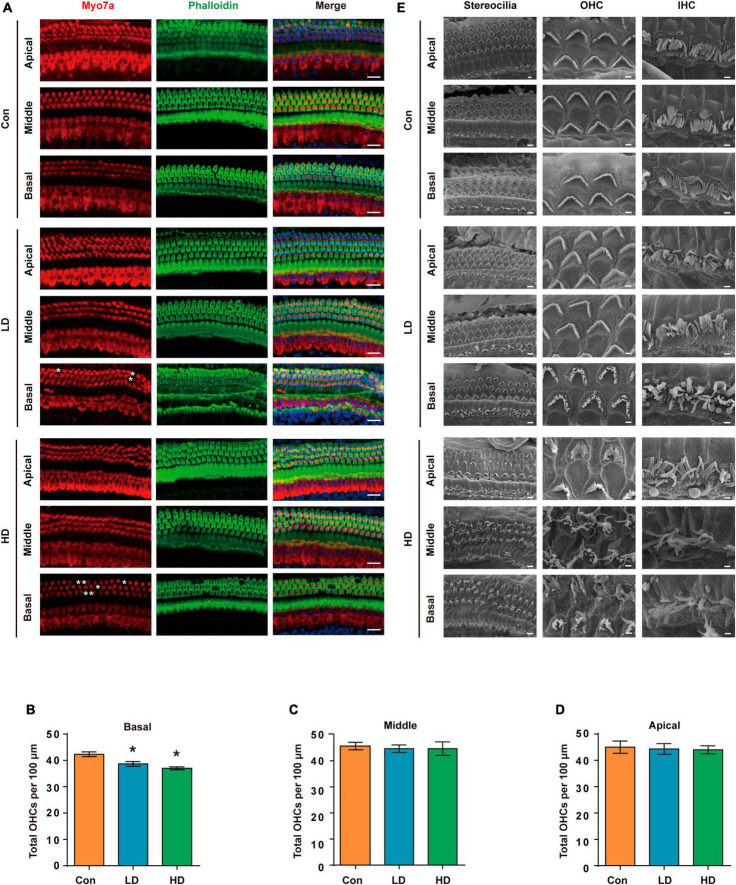
Histomorphological changes in cochlea following exposure to PS-NPs. **(A)** Examination of hair cell conditions at the apical, middle, and basal regions of the cochleae in control, LD, and HD group mice. Whole cochlear mounts were labeled with phalloidin for filamentous actin (green), myosin 7a (red) for hair cells, and DAPI (blue) for nuclei. Scale bar = 20 μm. **(B–D)** Quantification of the total number of OHCs per 100 μm cochlear length in control, LD, and HD group. Error bars represent the SEM. **(E)** Representative global view and magnification images of the stereocilia of cochlear hair cells in control, LD, and HD group mice shown by SEM. The scale bar in global view images is 4 μm, scale bar in magnification images is 1 μm. *p < 0.05, compared to control group. Con: control group, 0 mg/kg PS-NPs; LD: low dose group, 5 mg/kg PS-NPs; HD: high dose group, 25 mg/kg PS-NPs.

To evaluate the effect of PS-NPs on auditory function in mice, pristine PS-NPs were used for 8 weeks to establish mouse models as shown in the flowchart (Figure 2A). To investigate whether PS-NPs could enter the cochlea, mice were orally administered fluorescent PS-NPs for 4 weeks. Confocal imaging showed that fluorescent PS-NPs were observed in the spiral ganglion (SGN), the Organ of Corti, and the stria vascularis (SV) of the cochlea (Figure 2B). Bioaccumulation of fluorescent PS-NPs was observed in the inner ear by an animal *in vivo* imaging system (Figure 2C). ABR is the most commonly utilized technique for assessing the auditory function in mouse. Our ABR results showed that the mice exhibited high-frequency hearing deficits 4 weeks after exposure to PS-NPs, which subsequently worsened at the next 4 weeks of testing (Figures 2D–H). The common reason behind sensorineural hearing loss is the injury of hair cells located in the Corti organ of the cochlear auditory sensory epithelium (Hinton et al., 2021). To investigate hair cell pathology caused by PS-NPs, we observed the hair cells of mice by experimental preparation of cochlear epithelia for hair cell staining, and we performed scanning electron microscopy observations of the cochlea stereocilia as well as H&E staining. The results revealed that significant loss of OHCs was observed in the basal turns of mice in the LD and HD groups of mice (Figures 3A–D). There was no significant difference in OHCs loss between LD and HD groups. We also conducted a comprehensive examination utilizing scanning electron microscopy to analyze the structural integrity of the cochlear stereocilia. The control mice exhibited the typical structure of stereociliary bundles that were well-organized arrangement on the top of the hair cells. In contrast, mice in the LD group displayed disorganized stereocilia of hair cells in the basal turn. More seriously, mice in the HD group displayed a considerably more severe disarray of stereocilia in their hair cells. The results of the scanning electron microscopy showed that the distinct morphology of the stereocilia was altered following exposure to PS-NPs (Figure 3E). This alteration was evident in a detailed microscopic examination of the tissue, where the stereocilia lost their characteristic W-shape and the well-organized staircase pattern became disrupted. H&E staining showed that exposure to PS-NPs resulted in loss of outer hair cells (OHCs), and there was no significant abnormality in spiral ganglion and stria vascularis (Supplementary Figure 1). These results indicate that following oral administration of PS-NPs in mice, PS-NPs entered into cochlea and induce hair cell degeneration, resulting in hearing loss.

### 3.3 Down-regulation of tight junction proteins following exposure to PS-NPs

**FIGURE 4:**
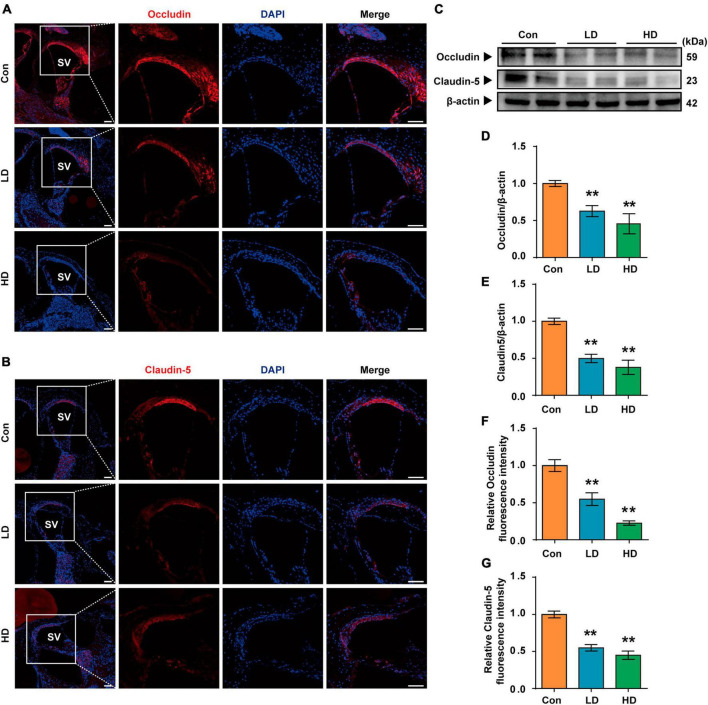
Expression of tight junction protein in BLB following the PS-NPs exposure. **(A,B)** Immunofluorescence analysis of Occludin and Claudin-5 in the cochlear stria vascularis, Scale bar = 200 μm for global images, scale bar = 50 μm for higher magnification. **(C)** Western blot analysis of Occludin and Claudin-5. **(D–G)** Quantification of WB and Immunofluorescence. Error bars represent SEM. **p < 0.01, compared to control group. Con: control group, 0 mg/kg PS-NPs; LD: low dose group, 5 mg/kg PS-NPs; HD: high dose group, 25 mg/kg.

Our research revealed that PS-NPs could accumulate in the cochlea following exposure to PS-NPs. However, the mechanism by which PS-NPs penetrate the blood-lymphatic barrier (BLB) in the mouse cochlea remains unclear. Existing evidence has indicated that PS-NPs exhibit the ability to be assimilated into the bloodstream via the gastrointestinal tract (Sun et al., 2022; Yang Z. S. et al., 2022). PS-NPs were detected in the bloodstream of mice in our study, which is consistent with the previous findings (Supplementary Figure 2). We further explored the effects of exposure of PS-NPs on the tight connections of PS-NPs exposure on the tight connection of BLB, and these effects exhibited a significantly lower expression of both Occludin and Claudin-5 in the LD and HD groups, as compared to the control group (Figures 4A–G).

### 3.4 Effects of PS-NPs exposure on cell apoptosis, oxidative stress, ferroptosis and inflammation in the cochlea

**FIGURE 5:**
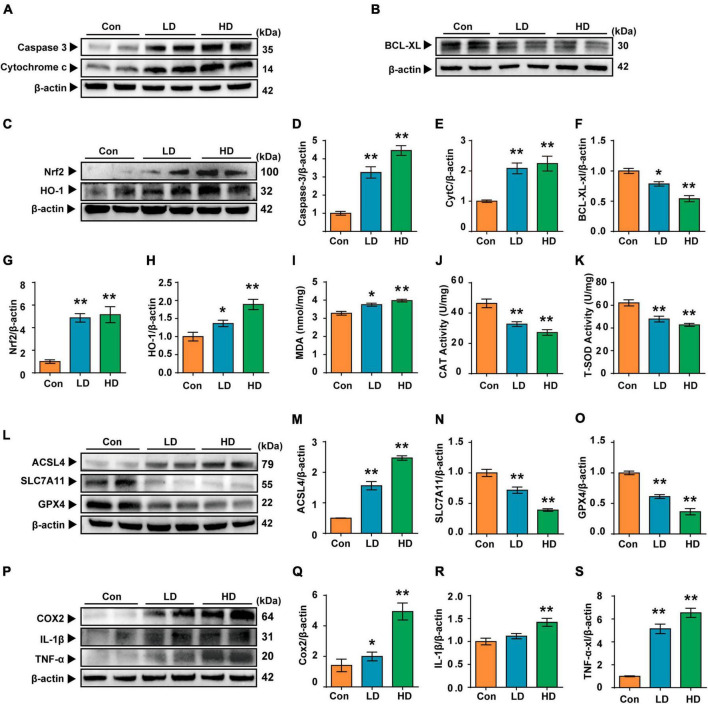
Polystyrene-NPs affect the expression of proteins related to apoptotic, oxidative stress, ferroptosis and inflammation in cochlea. **(A,B)** Western blot analysis was conducted to detect the protein expression of caspase 3, Cytochrome c and Bcl-xl. **(C)** The protein expression of Nrf2 and HO-1 was assessed using Western blotting. **(I–K)** The impact of PS-NPs on the activities of MDA and CAT, as well as the SOD content in mouse cochlear tissue, was evaluated. **(L,P)** Western blot analysis of ACSL4, SLC7A11, GPX4, COX2, IL-1β, TNF-α. **(D–H,M–O,Q–S)** Densitometric analysis was performed to present the results in a graphical form. Error bars represent SEM. *p < 0.05, **p < 0.01, compared to control group. Con: control group, 0 mg/kg PS-NPs; LD: low dose group, 5 mg/kg PS-NPs; HD: high dose group, 25 mg/kg.

In this study, we investigated the molecular mechanisms of cochlea injury induced by PS-NPs. We examined Caspase 3, Cytochrome c and BCL-XL levels to determine whether exposure to PS-NPs led to cell apoptosis in the cochlea. We found that the expression levels of Caspase 3 and Cytochrome c were increased (Figures 5A, D, E), and that BCL-XL exhibited a significant decrease in the LD and HD groups (Figures 5B, F). Next, we observed that the expression of NRF2 and HO-1 was increased in the cochlea following exposure to PS-NPs (Figures 5C, G, H).

Excessive ROS oxidizes lipids to induce lipid peroxidation (LPO). The MDA levels in cochlea exposed to PS-NPs were significantly higher than those in the control group, suggesting the occurrence of LPO in cell membranes (Figure 5I). In addition, excessive ROS can be evaluated by measuring antioxidant enzyme levels. In the current study, we observed significant inhibition of CAT and SOD in the cochlea exposed to PS-NPs (Figures 5J–K). This may be a response to excess ROS stimulated by PS-NPs in cells. Considering that ROS is closely related to ferroptosis signaling pathways in the process of cell death, we then detected the expression of ferroptosis signaling pathway-related indicators. In this research, the expression of GPX4 and SLC7A11 was found to be down-regulated (Figures 5L–O) and the expression of ACSL4 was significantly increased in the cochlea following exposure to PS-NPs (Figures 5L, M). Subsequently, we further detected the levels of pro-inflammatory cytokines including TNF-α, IL-1β and COX2. These cytokines are an important part of the immune system involved in the inflammatory response. Our results showed that exposure to PS-NPs increased the expression of pro-inflammation cytokines including TNF-α, IL-1β, and COX2 (Figures 5P–S). Our results thus suggested that cell apoptosis, oxidative stress, ferroptosis and inflammatory are involved in the ototoxicity induced by PS-NPs in the cochlea. These findings were consistent with the results of immunofluorescence staining (Supplementary Figure 3).

### 3.5 Entry of PS-NPs into HEI-OC1 cells and reduction of the viability of HEI-OC1 cells

**FIGURE 6:**
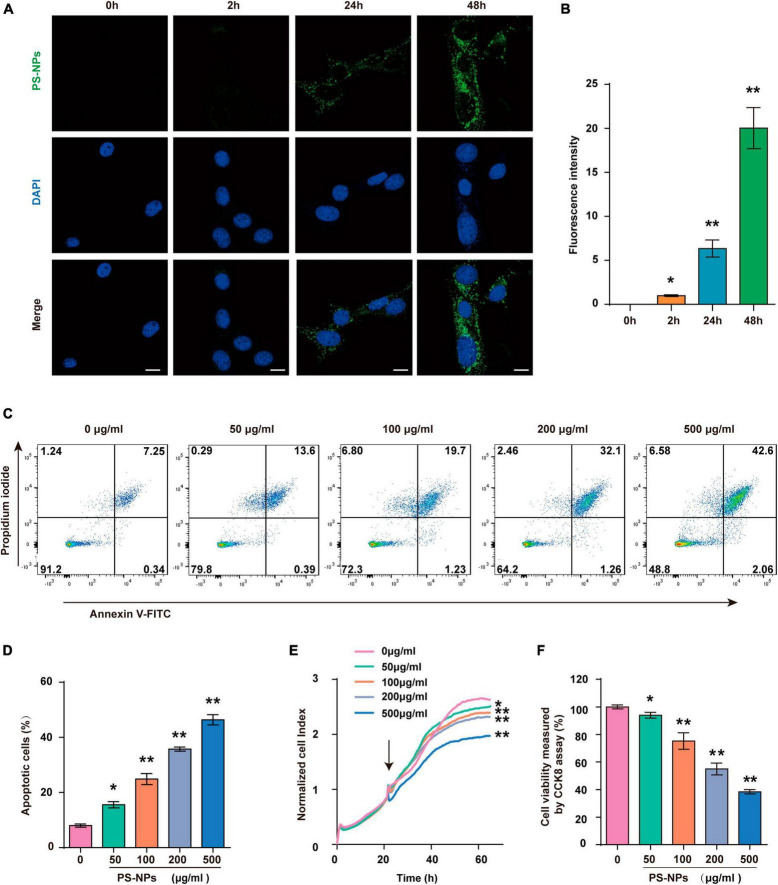
Polystyrene-NPs enter into HEI-OC1 cells and reduce the viability of HEI-OC1 cells. **(A,B)** The presence of fluorescent PS-NPs in HEI-OC1 cells treated with fluorescent PS-NPs after 0, 2, 24, and 48 h (Scale bar = 50 μm), the green color indicates PS-NPs, and the nuclei are blue (stained by DAPI), and the green fluorescence intensity of PS-NPs is quantified. **(C,D)** Flow cytometry analysis of apoptosis by the Annexin V-FITC/PI double staining assay after HEI-OC1 cells co-treated with different concentrations of fluorescent PS-NPs for 24 h and cell apoptotic rate is quantified. **(E)** RTCA shows that different concentrations of PS-NPs could reduce the viability of HEI-OC1 cells, and high concentrations of PS-NPs lead to more severe cell death. **(F)** HEI-OC1 cells treated with different concentrations of PS-NPs for 24 h are subjected to CCK-8 assay. Error bars represent SEM. *p < 0.05, **p < 0.01, compared to the control group.

In this study, we next used HEI-OC1 cells to further investigate PS-NPs ototoxicity and its underlying mechanism. Our results showed that the presence and intensity of fluorescent PS-NPs in HEI-OC1 cells after 2, 24, and 48-h treatment showed a time dependent pattern (Figures 6A, B). Further examination utilizing RTCA (Figure 6E) and CCK-8 (Figure 6F) demonstrated that the viability of HEI-OC1 cells was diminished following treated with pristine PS-NPs for 24 h, leading to a decrease in cell survival as the concentration of pristine PS-NPs increased. Based on the CCK-8 results, we calculated that the inhibitory concentration 50 (IC50) of pristine PS-NPs was 214.5 μg/mL. These findings supported the notion that the cytotoxicity of pristine PS-NPs is concentration-dependent, as reported in prior studies (Yang et al., 2021). Our results suggest that PS-NPs could enter into HEI-OC1 cells and adversely affect their viability.

### 3.6 Effects of exposure to PS-NPs on cell apoptosis, oxidative stress, ferroptosis, and inflammation in HEI-OC1 cells

**FIGURE 7:**
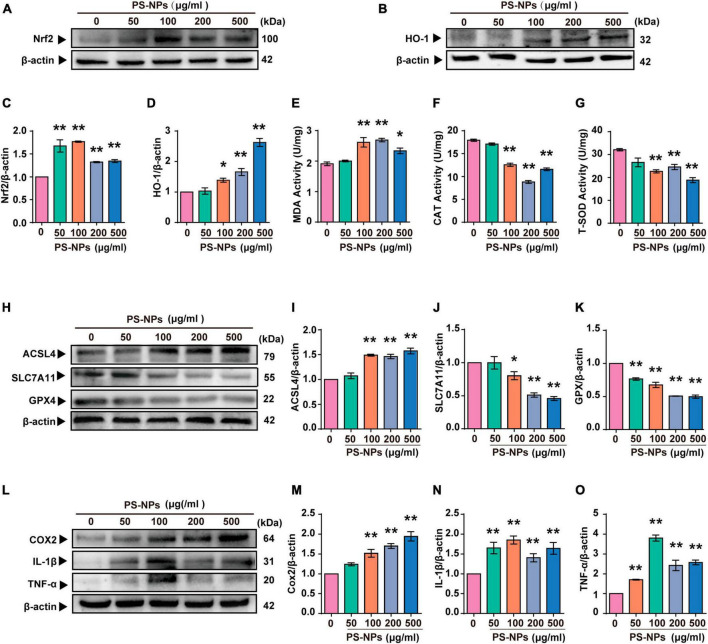
Polystyrene-NPs affect the expression of proteins related to oxidative stress, ferroptosis and inflammation in HEI-OC1 cells. **(A–D)** The protein expression of Nrf2 and HO-1 was assessed using Western blotting and densitometric analysis. **(E–G)** The impact of PS-NPs on the activities of SOD and CAT, as well as the MDA content in HEI-OC1 cells, were evaluated. **(H–O)** Western blot analysis of ACSL4, SLC7A11, GPX4, COX2, IL-1β, TNF-α and quantification of WB intensities. Error bars represent SEM. *p < 0.05, **p < 0.01, compared to the control group.

Similar to the experiments with mice, we examined the effects of different concentrations of PS-NPs on apoptosis, oxidative stress, ferroptosis, and inflammation-related indicators in HEI-OC1 cells. The Annexin V-FITC/PI double staining assay provided evidence that HEI-OC1 cell death was induced by PS-NPs through apoptosis, and the degree of apoptosis dependent on the concentration of PS-NPs (Figures 6C, D). Our *in vitro* experiments also confirmed that compared to the control group, SOD and CAT were significantly decreased, while Nrf2, HO-1, MDA levels were significantly increased following PS-NPs treatment. This proved the contribution of PS-NPs to oxidative damage (Figures 7A–G). Furthermore, increased expression of ferroptosis-related protein ACSL4 and decreased expression of SLC7A11 and GPX4 in HEI-OC1 cells following exposure to PS-NPs were also found *in vitro* (Figures 7H–K). Interestingly, our study also found that the content of the inflammatory factors TNF-α, IL-1β, and COX2 was increased (Figures 7L–O). Therefore our *in vitro* results also confirmed that apoptosis, oxidative stress, ferroptosis and inflammation were involved in the molecular mechanism of PS-NPs ototoxicity. This finding is consistent with our *in vivo* findings in the cochlea.

**FIGURE 8:**
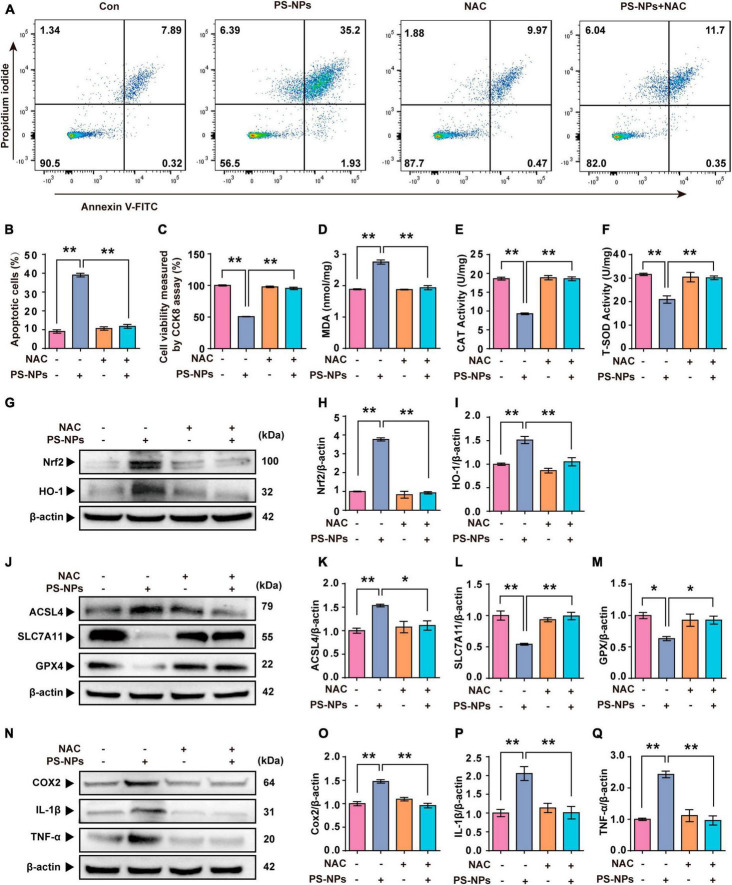
N-acetylcysteine inhibits PS-NPs-induced apoptotic, oxidative stress, ferroptosis and inflammation in HEI-OC1 cells. **(A,B)** Flow cytometry analysis of apoptosis by the Annexin V-FITC/PI double staining assay in HEI-OC1 cells exposed to PS-NPs with NAC pretreatment, and apoptotic rate is quantified. **(C)** Results of cell viability determination by CCK-8 assay. **(D–F)** The impact of PS-NPs on the activities of MDA and CAT, as well as the SOD content in HEI-OC1 cells with NAC pretreatment, are evaluated. **(G–Q)** Western blot analysis of Nrf2, HO-1, ACSL4, SLC7A11, GPX4, TNF-α, IL-1β, COX2 and quantification of WB intensities. Error bars represent SEM. *p < 0.05, **p < 0.01, compared to the control group.

**FIGURE 9:**
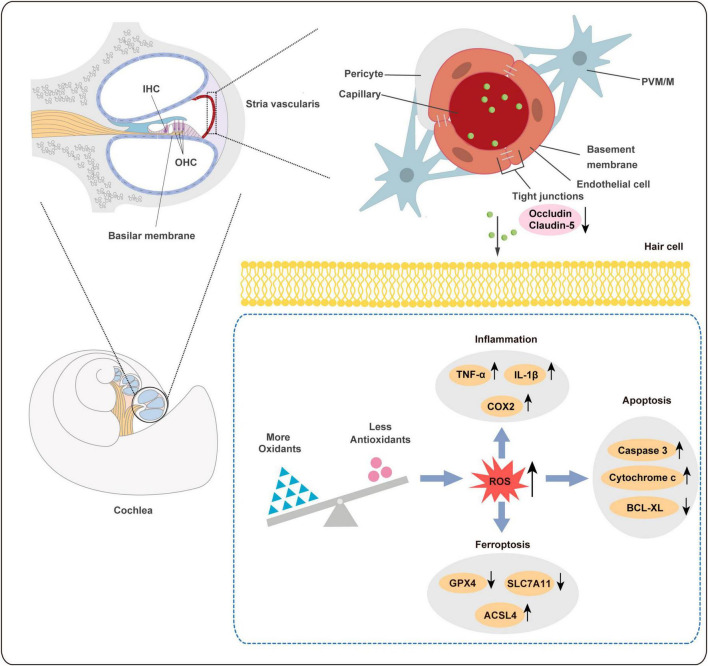
Schematic diagram of ototoxicity mechanism of PS-NPs. PS-NPs could enter the stria vascularis capillaries from the blood circulation, and then enter the cochlear hair cells through the BLB barrier, thereby causing cell apoptosis, ferroptosis, and inflammation in the hair cells. BLB: blood-lymphatic barrier.

In our study, NAC was used as an antioxidant to investigate the key role of ROS in PS-NPs induced molecular pathological changes in HEI-OC1 cells. NAC treatment of HEI-OC1 cells resulted in a reversal of cell apoptosis and a decrease of cell activity induced by PS-NPs (Figures 8A–C). As noted above, NAC significantly reduced the expression of Nrf2, HO-1, and MDA, and also reversed the decrease of SOD and CAT. This finding indicates that NAC reduced the oxidative damage induced by PS-NPs (Figures 8D–I). We also found that NAC alleviated the reduction in GPX4 and SLC7A11 protein expression, and also reversed the increase of ACSL4 (Figures 8J–M). We further examined inflammatory factors and found that the levels of TNF-α, IL-1β, and COX2 in HEI-OC1 cells following NAC treatment were significantly decreased (Figures 8N–Q). These results suggest a critical role for ROS in cell apoptosis, ferroptosis, and inflammation induced by PS-NPs. We created a schematic diagram to elucidate the ototoxicity mechanism of PS-NPs (Figure 9).

### 3.7 PS-NPs cause a decrease in the acoustic startle response capacity of the zebrafish by destroying the lateral line hair cells

**FIGURE 10:**
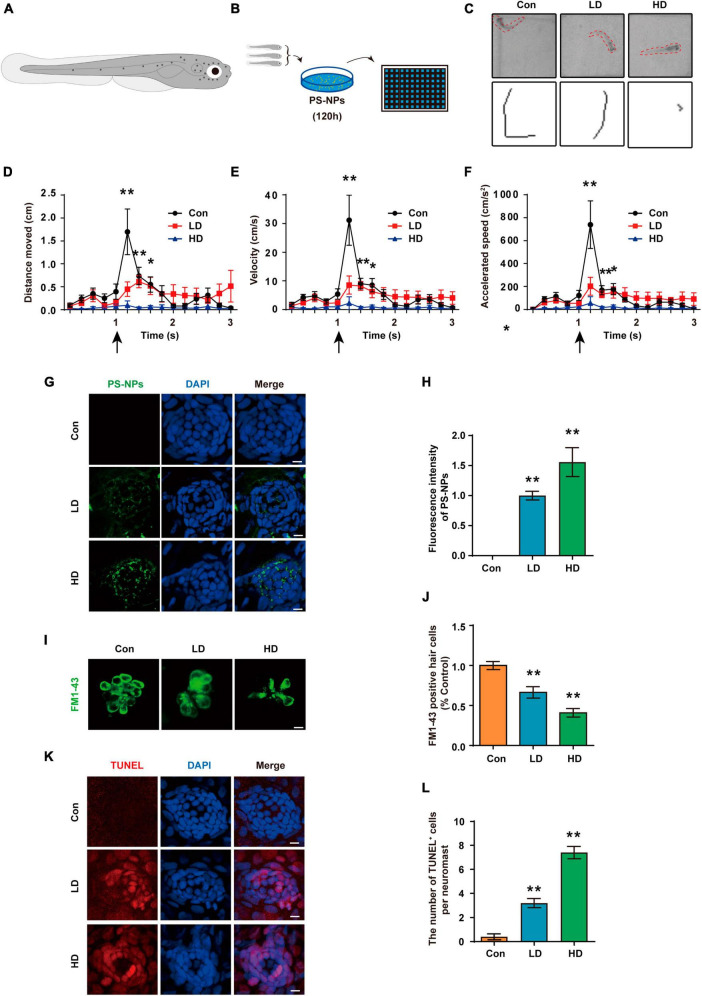
Effects of PS-NPs on auditory function and lateral line neuromast hair cells in zebrafish. **(A)** Diagram of a 5 dpf zebrafish larvae. **(B)** Zebrafish (5 dpf) are placed in EM containing PS-NPs for 120 h. **(C)** Characteristic C-bend motion identified in a single picture frame from a subset of zebrafish and moving traces identified from multiple picture frames after delivering a stimulus. **(D–F)** Startle response of zebrafish with PS-NPs treatment of different level of concentration. Mean moving distance, velocity, and acceleration are used as the quantification parameters after sound stimulation. **(G)** The presence of fluorescent PS-NPs in lateral line neuromasts cell co-treated with different concentrations PS-NPs after 120 h. The green color indicates PS-NPs, and the nuclei are blue (stained by DAPI). **(H)** The green fluorescence intensity of PS-NPs is quantified. **(I)** Lateral line hair cells stained with FM1-43 (green). **(J)** The FM1-43 positive cells counting is quantified. **(K)** Immunofluorescence staining for TUNEL (red) in neuromasts after treatment with PS-NPs. **(L)** The number of TUNEL positive cells per neuromast cell. Scale bar = 5 μm. Error bars represent SEM. *p < 0.05, **p < 0.01, compared to control group. Con: control group, 0 μg/mL PS-NPs; LD: low dose group, 0.005 mg/mL PS-NPs; HD: high dose group, 0.05 mg/mL.

Acoustic startle response has become a crucial behavioral method to evaluate the function of lateral line system (Yang et al., 2017; Liu et al., 2018). We experimented with 5 dpf zebrafish (Figure 10A). Zebrafish are placed in EM containing PS-NPs for 120 h (Figure 10B). In this study, the acoustic startle response test of zebrafish was measured by the C-type bending response of the zebrafish body, as well as the distance of movement, maximum speed and maximum acceleration. Our results showed that the C-type bending response of zebrafish in LD and HD groups was significantly lower than the response of control zebrafish (Figure 10C). Further, quantitative analysis showed that the distance of movement, velocity, and acceleration speed of zebrafish in the LD and HD groups were also significantly lower than that in the control (Figures 10D–F). Our results confirmed that PS-NPs cause a decrease in the acoustic startle response capacity in zebrafish.

We observed that fluorescent PS-NPs aggregated in lateral neuromasts through confocal fluorescence localization. This indicates that PS-NPs are able to be absorbed by lateral neuromasts (Figures 10G, H). To further reveal the mechanism by which PS-NPs caused the decrease of acoustic startle response in zebrafish, hair cells were visualized by FM1-43 staining. Our experiment confirmed a reduction in the number of FM1-43-positive cells after zebrafish exposure to PS-NPs, particularly in the HD group (Figures 10I, J). To further investigate the effects of PS-NPs on lateral line neuromast cells in zebrafish, we labeled apoptotic cells with TUNEL. The results showed that compared with the control group, the number of TUNEL-positive cells in the LD and HD groups increased significantly, especially in the HD group (Figures 10K, L). These results indicated that PS-NPs damaged zebrafish lateral line hair cells.

## 4 Discussion

Polystyrene-NPs, as a novel pollutant, are widely distributed in water, land, and atmospheric environments, raising concerns about their potential threats to organisms, particularly mammals and aquatic organisms. Existing evidence has demonstrated that PS-NPs accumulate in several organs of mice, including the uterus, blood, ovaries, heart, liver, spleen, lungs, kidneys, small and large intestines, testes, and brain (Xu et al., 2021; Liu et al., 2022). However, there is currently insufficient knowledge regarding the accumulation and distribution of PS-NPs in cochlea. Our study conducted a systematic examination of the ototoxic effects of PS-NPs on mammals and aquatic organisms for the first time, using mice, HEI-OC1 cells, and zebrafish.

Given the protective effect of estrogen on hearing, there may be sex differences in the toxicity of PS-NPs (Delhez et al., 2020; Villavisanis et al., 2020), we only used male mice in this study. The PS-NPs used in this study were in the range of environmental size (Pivokonsky et al., 2018; Ruan et al., 2024). The dose of LD group in mouse experiments was within the range of environmental concentrations reported by Liang et al. (2021) and Senathirajah et al. (2021). Higher doses were required to observe lateral line functional impairment in zebrafish for practical study reasons (Shi et al., 2024).

In general, the BLB is a dense network of capillaries located in the stria vascularis, and it is a sandwich of epithelial marginal cells and mesodermal basal cells that are connected by tight junctions (TJ). Dysregulation of TJ proteins may lead to the disruption of BLB (Liu et al., 2013). The endothelium of the BLB is encompassed by a basement membrane, which is further enveloped by pericytes that enclose the capillaries. Additionally, the basement membrane surrounding the BLB endothelium is covered by perivascular-resident macrophage-like melanocytes (PVM/Ms) (Nyberg et al., 2019). BLB has a similar function as the blood-brain barrier (BBB) and is linked to the endothelial cell transport system, which contains TJ. Studies have found that PS-NPs exposure can disrupt the expression of certain TJ proteins and increase the permeability in the BBB (Lee et al., 2022; Yin et al., 2022). In addition, previous studies have shown that Claudin-5 and Occludin are involved in the ultrastructure changes, increased permeability, and hearing loss caused by noise and lead-induced BLB damage (Liu et al., 2013; Wu et al., 2014). Our results revealed that the expression of Occludin and Claudin-5 proteins was significantly reduced following exposure to PS-NPs. Therefore, PS-NPs may impair TJ by down-regulating Occludin and Claudin-5 expression, potentially affecting BLB integrity, and allowing PS-NPs to penetrate the BLB and reach the cochlea.

Previous studies have demonstrated that hair cell apoptosis can be triggered by several types of damage such as toxicity, noise exposure, and aging (Op de Beeck et al., 2011). Our study obtained similar results as above. Nrf2 plays a crucial role in regulating the expression of genes involved in antioxidant defense and detoxification (Tonelli et al., 2018). Under physiological conditions, cytosolic thiol antioxidants maintain the stability of inactive KEAP1/Nrf2 heterodimers. When the cytosolic thiol levels are diminished by ROS, the heterodimer becomes dissociated, and Nrf2 translocates to cell nucleus, where it binds to antioxidant response elements (ARE) in the form of Nrf2-Maf. This promotes downstream antioxidant gene transcription such as HO-1, CAT, SOD (Chen et al., 2020). Excessive ROS oxidizes lipids to induce LPO, which can be evaluated by measuring antioxidant enzyme levels. In this study, the levels of MDA in the cochlea and HEI-OC1 cells following exposure to PS-NPs were significantly increased, suggesting the presence of LPO in cell membranes. Additionally, we observed significant inhibition in SOD and CAT in the cochlea and HEI-OC1 cells following exposure to PS-NPs, which may be a response to excess ROS stimulated by PS-NPs in cells.

In recent years, increasing studies have revealed that ROS is closely related to ferroptosis. Ferroptosis is a novel type of iron-dependent cell death resulting from the lethal accumulation of lipid-based ROS. GPX4 has been shown to play a crucial role in protecting cells from ferroptosis caused by membrane LPO (Kang et al., 2019). System Xc^–^ is a heterodimer composed of a light chain subunit (SLC7A11) and a heavy chain subunit (SLC3A2), and down-regulation of SLC7A11 could indirectly inhibit the activity of GPX4 by inhibiting the cysteine metabolism pathway. This leads to a decrease in intracellular cysteine levels and depletion of glutathione biosynthesis, resulting in lipid peroxide accumulation and ultimately inducing cell ferroptosis (Koppula et al., 2021). Furthermore, ACSL4 is the key enzyme in the synthesis of polyunsaturated acyl tails and studies have demonstrated that it enhances cell sensitivity to ferroptosis by enriching membranes for specific oxidation-sensitive fatty acids. Therefore, ACSL4 is now considered a reliable biomarker of ferroptosis (Chen et al., 2021).

Several genes and pathways associated with ferroptosis have been identified (Jiang et al., 2021). Furthermore, research has recently found that ferroptosis is involved in organ toxicity induced by PS-NPs, including the small intestine, lungs, and kidneys (Qiu et al., 2023; Tang et al., 2023; Wu et al., 2023). In our research, the expression of GPX4 and SLC7A11 was significantly decreased and the expression of ACSL4 was significantly increased in cochlea and HEI-OC1cells following exposure to PS-NPs. Our results suggested that ferroptosis was also involved in ototoxicity induced by PS-NPs in the cochlea and HEI-OC1 cells.

In general, redundant ROS leads to oxidative stress and depletion of intracellular antioxidants, further exacerbating LPO production and inflammatory responses, and thus creating a vicious cycle (Yu et al., 2021). Excessive ROS is capable of activating transcription factors including pro-inflammatory cytokines like IL-1β and TNF-α, leading to progression of inflammation (Linkermann et al., 2014). Studies have demonstrated that MPs increased the expression of inflammatory factors IL-6, IL-1β, and TNF-α in mouse testicular tissue (Xie et al., 2020; Hou et al., 2021). Another study reported that PS-NPs induced myocardial inflammation in carp by promoting ROS production (Wu et al., 2022). Recent research found that maternal exposure to PS-NPs during pregnancy and breastfeeding led to inflammatory cell infiltration and up-regulation of pro-inflammatory cytokines in the liver of male mouse pups (Huang et al., 2022). Corresponding with the above results, our study found a similar inflammatory response induced by PS-NPs.

Reactive oxygen species are signaling molecules that are essential for response to stress and cellular signaling (Villalpando-Rodriguez and Gibson, 2021). However, overabundance of ROS leads to initiation of apoptosis and ferroptosis and further contributes to tissue impairment (Su et al., 2019). NAC is frequently utilized as a ROS inhibitor in scientific research. Its application has been instrumental in determining the significant role of ROS in cell apoptosis, ferroptosis pathways, and inflammatory response. Sun et al. reported that NAC alleviated the inflammatory response and ferroptosis of BV2 microglia cells induced by PS-NPs (Sun et al., 2023). Li et al. (2023) found that mitochondrial damage, cell apoptosis, and oxidative stress in human liver HepG2 cells induced by PS-NPs were reversed by pretreatment of NAC. HEI-OC1 cell line serves as an effective tool for investigating the potential mechanism of ototoxicity associated with these nanoparticles. As we had hypothesized, NAC treatment of HEI-OC1 cells resulted in a reversal of the decrease of cell activity as well as cell apoptosis, ferroptosis and inflammatory response induced by PS-NPs. Thus, these results showed that the participation of ROS plays a crucial role in the mechanism of ototoxicity induced by PS-NPs.

Previous studies have shown that fish ingest PS-NPs in aquatic environments, but the impact of PS-NPs on hair cells in the lateral line system of zebrafish has not been studied (Garcia-Torne et al., 2022). For the first time, we evaluated the toxicity of PS-NPs to lateral hair cells of zebrafish. Our results provide evidence that PS-NPs could accumulate and produce cytotoxicity in lateral line hair cells of zebrafish larvae. None of the concentrations used in this study were lethal, although we did not keep treated fish for long periods to observe potential long-term effects of PS-NPs treatment.

Our 2-month study provides valuable insights into short-term ototoxicity, but long-term effects remain unclear. *In vitro* results (Figure 6E) and the age-dependent hearing loss in mice (Figures 2E–H) suggest time-dependent effects. Preliminary human data (Schwabl et al., 2019) provided evidence of the excretion these plastic particles, complicating predictions regarding permanent or transient hearing loss. Considering the long-term existence of environmental pollution, further research with longer durations is crucial to fully understand the long-term impact of PS-NPs on hearing.

It is worth noting that in order to avoid the interference of fluorescent substances on experimental results, we only used pristine PS-NPs for toxicity assessment, while fluorescent PS-NPs were only used to observe and locate the distribution of PS-NPs in tissues and cells. In summary, this study conducted a preliminary examination of the ototoxic effects of PS-NPs on mammals and aquatic organisms for the first time, using mice, HEI-OC1 cells, and zebrafish. Although our current work has preliminarily revealed ototoxicity and its mechanism, this is only the tip of the iceberg that calls for further study on PS-NPs and their role in ototoxicity.

## 5 Conclusion

This study first of all discovered and systematically revealed the accumulation and toxicity of PS-NPs on the auditory organ and cells. Our investigation revealed that PS-NPs could increase BLB permeability by disrupting tight junctions, leading to the absorption of PS-NPs in the cochlea. The accumulation of PS-NPs in hearing organs can produce ototoxicity, which could lead to hearing loss. Furthermore, ROS may play a significant role in cell apoptosis, oxidative stress, ferroptosis, and inflammation triggered by PS-NPs, which are involved in the ototoxicity of PS-NPs. To our knowledge, these findings provide new insights into the toxicity of PS-NPs and call for a thorough assessment of the hearing health risks of PS-NPs to auditory health and emphasize the need for further research in this area to better understand and mitigate the hazards associated with PS-NPs exposure in humans.

## Data availability statement

The raw data supporting the conclusions of this article will be made available by the authors, without undue reservation.

## Ethics statement

The animal study was approved by the Animal Use and Care Committee of Binzhou Medical University. The study was conducted in accordance with the local legislation and institutional requirements.

## Author contributions

YW: Writing – original draft. LL: Writing – review and editing. LT: Formal Analysis, Writing – review and editing. WP: Writing – review and editing. HZ: Methodology, Writing – review and editing. DX: Methodology, Writing – review and editing. RG: Writing – review and editing. TZ: Methodology, Writing – review and editing. LB: Methodology, Writing – review and editing. XW: Methodology, Writing – review and editing. H-JC: Writing – review and editing. LW: Writing – review and editing. LZ: Methodology, Writing – review and editing. BL: Resources, Writing – original draft. QZ: Resources, Writing – review and editing.
