# Supplementary material for: Ototoxicity of polystyrene nanoplastics in mice, HEI-OC1 cells and zebrafish
Source: Front Mol Neurosci. 2024 Feb 19;17:1345536. doi: 10.3389/fnmol.2024.1345536 (PMC10909942; doi:10.3389/fnmol.2024.1345536)
Supplement: Supplementary file 1 [file Data_Sheet_1.DOCX]

Supplementary Information

*1.1. Hematoxylin-Eosin (H&E) staining of cochlea.*

The cochleae of mice were immersed in 4% paraformaldehyde for fixation, which was carried out for 24 hours at 4°C. Subsequently, the tissues were decalcified using a 10% EDTA solution for a period of three days before being embedded in paraffin. Tissue sections measuring 5 μm were stained with H&E and observed under an optical microscopy (DMI4000 B, Leica, Germany).

*1.2. Detection of fluorescent PS-NPs in the blood of mice.*

To verify PS-NPs bead presence in blood, confocal laser scans are conducted. A blood sample of 10 µl is collected from the mouse's tail vein and smears are prepared. Further detection of the blood sample is carried out with confocal microscopy.


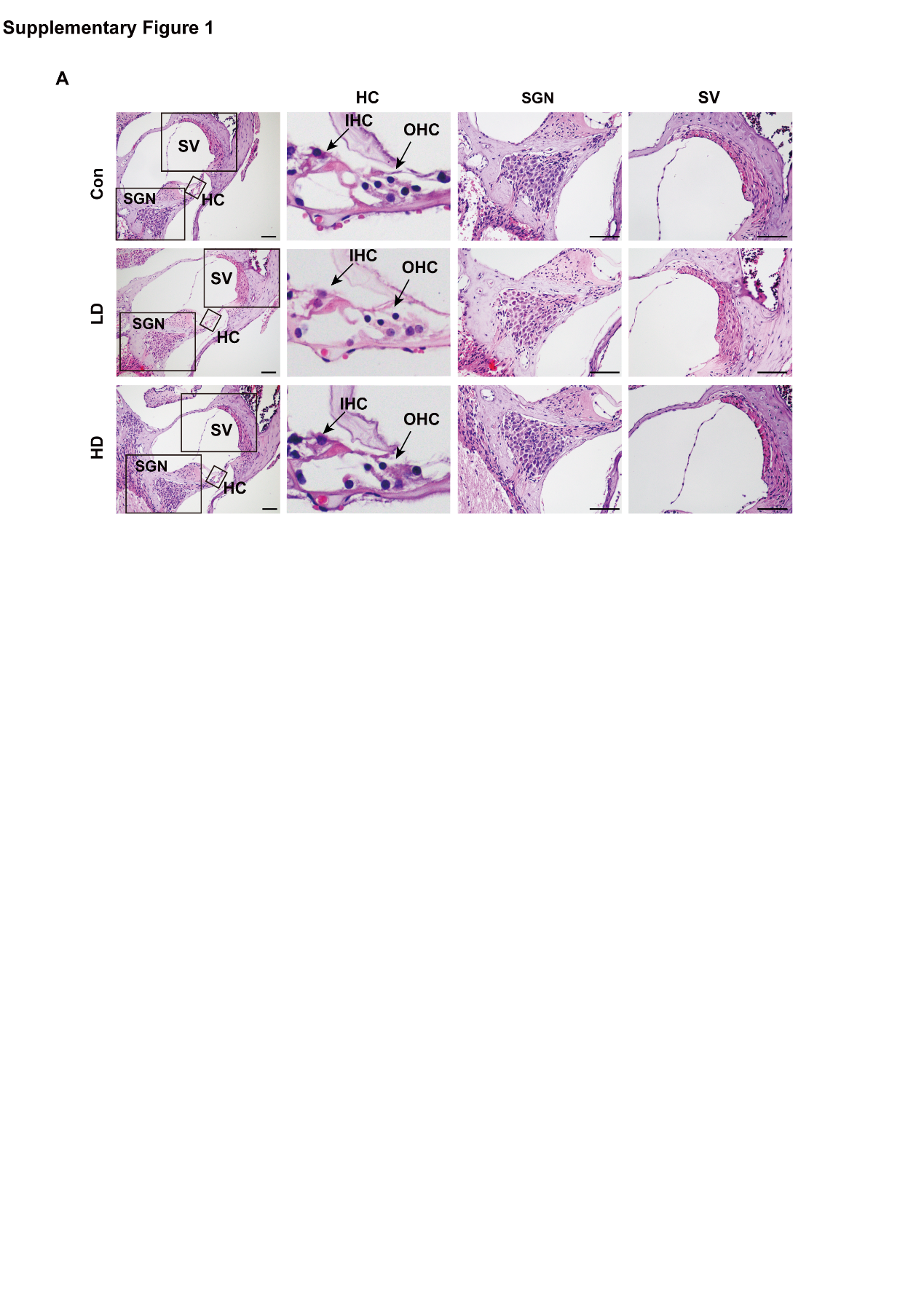


Figure S1. Morphological observation by H&E staining of the cochlea of mice. (A) HC, SGN, and SV in the cochlea were observed by H&E staining. HC: hair cell; IHC: inner hair cell; OHC: outer hair cells; SGN: [spiral ganglion](javascript:;); SV: [stria vascularis](javascript:;); Con: control group, 0 mg/kg PS-NPs; HD: high dose group, 25 mg/kg.


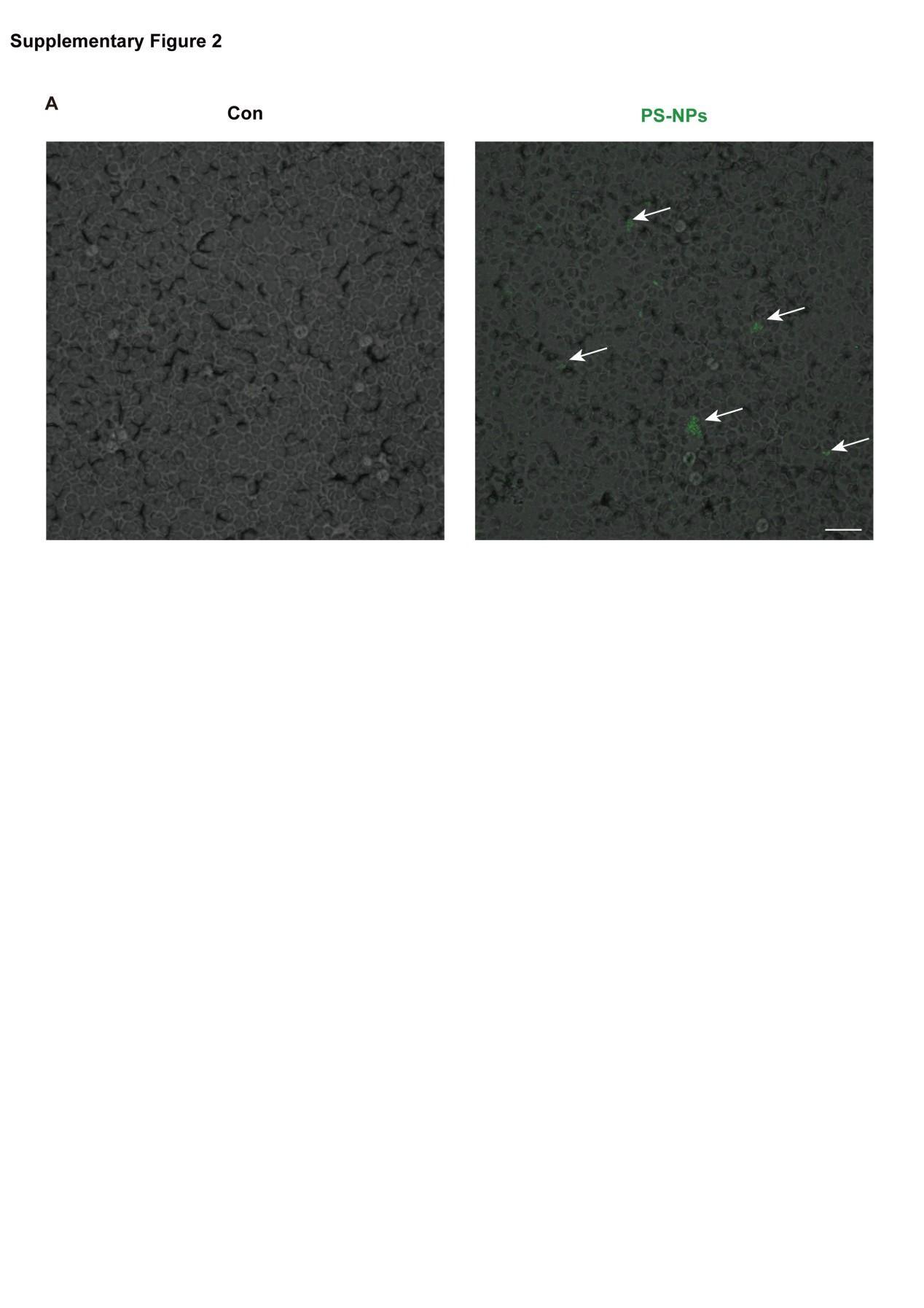


**Figure S2. The evidence of** **the presence of fluorescent PS-NPs in blood samples examined by confocal microscopy.** (A) Blood smears were used to observe fluorescent PS-NPs in the blood by confocal microscopy. [The arrows point to](javascript:;) fluorescent PS-NPs. Scale bar = 50 µm.


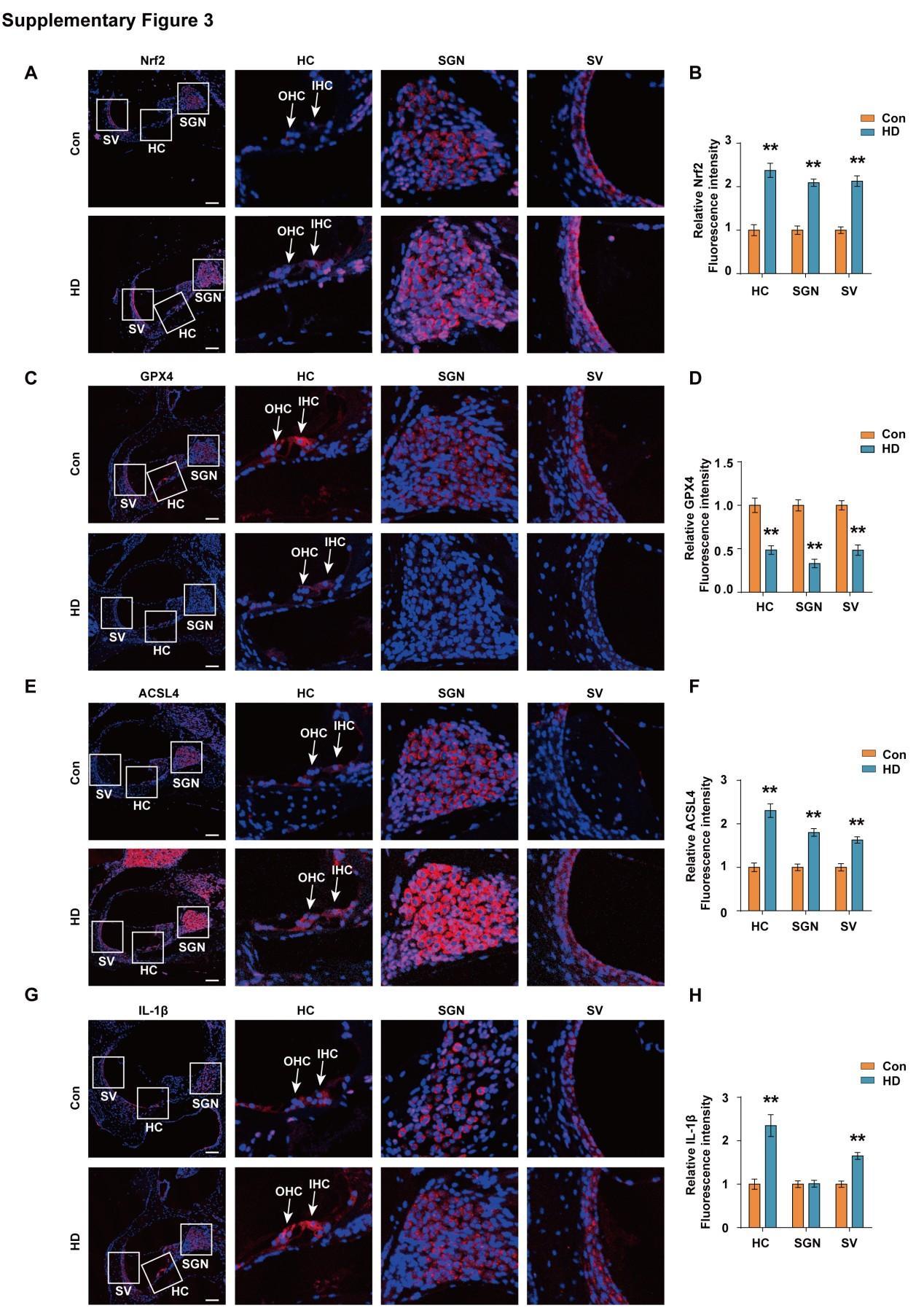


**Figure S3. [Immunofluorescence analysis](javascript:;) of Nrf2, GPX4, ACSL4 and IL-1β in cochlea of mice.** (A-B) Immunofluorescent staining of Nrf2 and relative fluorescent light intensity. (C-D) Immunofluorescent staining of GPX4 and relative fluorescent light intensity of. (E-F) Immunofluorescent staining of ACSL4 and relative fluorescent light intensity of. (G-H) Immunofluorescent staining of IL-1β and relative fluorescent light intensity. Scale bar = 200 µm. Error bars represent SEM. Compared to control group; **p* < 0.05, ***p* < 0.01. HC: hair cell; IHC: inner hair cell; OHC: outer hair cells; SGN: [spiral ganglion](javascript:;); SV: [stria vascularis](javascript:;); Con: control group, 0 mg/kg PS-NPs; HD: high dose group, 25 mg/kg.

**Supplementary Table 1**

**Table S1 Description and list of antibodies**

| **Antibody** | **Supplier** | **Cat#** | **Species** | **RRID** | **Dilution** |
| --- | --- | --- | --- | --- | --- |
| Goat Anti-Rabbit IgG H&L (HRP) | Abcam | ab6721 | Goat polyclonal | AB_955447 | 1:5000 |
| [Goat Anti-Mouse IgG H&L (HRP)](https://www.abcam.cn/products/secondary-antibodies/goat-mouse-igg-hl-hrp-ab6789.html) | Abcam | Ab6789 | Goat polyclonal | AB_955439 | 1:5000 |
| Goat anti-Rabbit IgG (H+L) Alexa Fluor™ 647 | Life Technologies | A-21245 | Goat polyclonal | AB_141775 | 1:500 |
| anti-Myosin 7a | Proteus BioSciences | 25-6790 | Rabbit polyclonal | AB_10015251 | 1:150 |
| anti-Nrf2 | Abcam | ab62352 | Rabbit monoclonal | AB_944418 | 1:1000 |
| anti-HO-1 | Abcam | ab52947 | Rabbit monoclonal | AB_880536 | 1:1000 |
| anti-Caspase 3 | [Cell Signaling Technology](http://www.baidu.com/link?url=CZ90QppqQZDC0UQtk-OkrCNSCy2_rGVLDQBeBnNmBP8-iWuyeM9zJ2B3X0ofIb29) | 9662 | Rabbit polyclonal | AB_331439 | 1:1000 |
| anti-[Cytochrome c](https://www.cellsignal.cn/products/primary-antibodies/cytochrome-c-136f3-rabbit-mab/4280?site-search-type=Products&N=4294956287&Ntt=cyc&fromPage=plp) | [Cell Signaling Technology](http://www.baidu.com/link?url=CZ90QppqQZDC0UQtk-OkrCNSCy2_rGVLDQBeBnNmBP8-iWuyeM9zJ2B3X0ofIb29) | 4272 | Rabbit polyclonal | AB_2090454 | 1:1000 |
| anti-[BCL-XL](https://www.cellsignal.cn/products/primary-antibodies/cytochrome-c-136f3-rabbit-mab/4280?site-search-type=Products&N=4294956287&Ntt=cyc&fromPage=plp) | [Cell Signaling Technology](http://www.baidu.com/link?url=CZ90QppqQZDC0UQtk-OkrCNSCy2_rGVLDQBeBnNmBP8-iWuyeM9zJ2B3X0ofIb29) | 2764 | Rabbit monoclonal | AB_2228008 | 1:500 |
| anti-[ACSL4](https://www.cellsignal.cn/products/primary-antibodies/cytochrome-c-136f3-rabbit-mab/4280?site-search-type=Products&N=4294956287&Ntt=cyc&fromPage=plp) | Abcam | ab155282 | Rabbit monoclonal | AB_2714020 | 1:1000 |
| anti-[SLC7A11](https://www.cellsignal.cn/products/primary-antibodies/cytochrome-c-136f3-rabbit-mab/4280?site-search-type=Products&N=4294956287&Ntt=cyc&fromPage=plp) | [Proteintech](http://www.ptgcn.com/) | 26864 | Rabbit polyclonal | AB_2880661 | 1:500 |
| anti-[GPX4](https://www.cellsignal.cn/products/primary-antibodies/cytochrome-c-136f3-rabbit-mab/4280?site-search-type=Products&N=4294956287&Ntt=cyc&fromPage=plp) | Abcam | Ab125066 | Rabbit monoclonal | AB_10973901 | 1:2000 |
| anti-[COX2](https://www.cellsignal.cn/products/primary-antibodies/cytochrome-c-136f3-rabbit-mab/4280?site-search-type=Products&N=4294956287&Ntt=cyc&fromPage=plp) | Abcam | Ab179800 | Rabbit monoclonal | AB_2894871 | 1:500 |
| anti-TNF-α | [Proteintech](http://www.ptgcn.com/) | 60291 | Mouse monoclonal | AB_2883456 | 1:1000 |
| anti-[IL-1β](https://www.cellsignal.cn/products/primary-antibodies/cytochrome-c-136f3-rabbit-mab/4280?site-search-type=Products&N=4294956287&Ntt=cyc&fromPage=plp) | [Proteintech](http://www.ptgcn.com/) | 16806 | Rabbit polyclonal | AB_10646432 | 1:500 |
| anti-β-actin | [Cell Signaling Technology](http://www.baidu.com/link?url=CZ90QppqQZDC0UQtk-OkrCNSCy2_rGVLDQBeBnNmBP8-iWuyeM9zJ2B3X0ofIb29) | 4970 | Rabbit monoclonal | AB_2223172 | 1:1000 |
